# Infectious Bursal Disease Virus VP5 Polypeptide: A Phosphoinositide-Binding Protein Required for Efficient Cell-to-Cell Virus Dissemination

**DOI:** 10.1371/journal.pone.0123470

**Published:** 2015-04-17

**Authors:** Fernando Méndez, Tomás de Garay, Dolores Rodríguez, José F. Rodríguez

**Affiliations:** Department of Molecular and Cellular Biology, Centro Nacional de Biotecnología-CSIC, Cantoblanco, 28049, Madrid, Spain; INRA, FRANCE

## Abstract

Infectious bursal disease virus (IBDV), a member of the *Birnaviridae* family, is a major avian pathogen responsible for an immunosuppressive disease affecting juvenile chickens. The IBDV genome is formed by two dsRNA segments. The largest one harbors two partially overlapping open reading frames encoding a non-structural polypeptide, known as VP5, and a large polyprotein, respectively. VP5 is non-essential for virus replication. However, it plays a major role in IBDV pathogenesis. VP5 accumulates at the plasma membrane (PM) of IBDV-infected cells. We have analyzed the mechanism underlying the VP5 PM targeting. Updated topological prediction algorithm servers fail to identify a transmembrane domain within the VP5 sequence. However, the VP5 polycationic C-terminal region, harboring three closely spaced patches formed by two or three consecutive basic amino acid residues (lysine or arginine), might account for its PM tropism. We have found that mutations, either C-terminal VP5 deletions or replacement of basic amino acids by alanine residues, that reduce the electropositive charge of the VP5 C-terminus abolish PM targeting. Lipid overlay assays performed with an affinity-purified Flag-tagged VP5 (FVP5) protein version show that this polypeptide binds several phosphoinositides (PIP), exhibiting a clear preference for monophosphate species. Experiments performed with FVP5 mutant proteins lacking the polycationic domain demonstrate that this region is essential for PIP binding. Data gathered with IBDV mutants expressing C-terminal deleted VP5 polypeptides generated by reverse genetics demonstrate that the VP5-PIP binding domain is required both for its PM targeting in infected cells, and for efficient virus dissemination. Data presented here lead us to hypothesize that IBDV might use a non-lytic VP5-dependent cell-to-cell spreading mechanism.

## Introduction

Infectious bursal disease virus (IBDV), the best characterized member of the *Birnaviridae* family, is the etiological agent of an immunosuppressive disease (IBD) that affects juvenile domestic chickens (*Gallus gallus*) [1]. The global importance of this disease is highlighted by its inclusion in the list of notifiable diseases by the World Organisation for Animal Health (OIE) (http://www.oie.int/en/animal-health-in-the-world/oie-listed-diseases-2014/).

The IBDV genome is formed by two dsRNA segments, A and B, respectively. Segment A harbors two open reading frames (ORF) coding the non-structural VP5 polypeptide, and the virus polyprotein, respectively [2]. The VP5 ORF overlaps the 5′ end region of the polyprotein ORF, thus transcription of this segment results in the synthesis of bicistronic mRNA molecules. The mechanism(s) regulating the translation of both ORFs remains uncharacterized. Bioinformatics analyses indicate that the VP5 ORF originated by gene overprinting on an ancestral polyprotein gene [3]. The presence of VP5 orthologs only in genomes from viruses belonging to two, i.e. avibirnavirus and aquabirnavirus, of the four birnavirus genera suggests that this ORF was generated late during birnavirus evolution.

Information concerning the specific role of VP5 during virus replication is somewhat controversial. Whilst some reports describe VP5 as a proapoptotic polypeptide [4,5], others provide experimental data supporting that it prevents IBDV-induced apoptosis [6,7]. According to several reports VP5 expression enhances the virus progeny size and promotes virus release [6,8,9].

Although VP5 expression is not essential for virus replication either in tissue culture or in experimentally infected chickens [4,8], VP5 knockout mutants are strongly attenuated, thus failing to induce clinical signs of disease and sizable bursal lesions [4,10]. This indicates that VP5 is a major IBDV virulence factor playing a key role in viral pathogenesis. Despite the enormous amount of work devoted to unravel molecular basis underlying IBDV pathogenesis our understanding about this critical issue is as yet scarce [11]. The IBDV protein arsenal is quite narrow, encompassing only five mature polypeptides (VP1-5). Although much effort has been focused on the characterization of structural polypeptides (VP1-3) and the virus-encoded protease (VP4), structural and functional data concerning VP5 is comparatively scarce. Indeed, an in depth knowledge about VP5 might provide new clues for the understanding of IBDV-host interactions.

VP5 localizes at the plasma membrane (PM) of infected cells [2,9,12]. In a previous report we suggested the possibility that VP5 might behave as a type II transmembrane polypeptide [12], however bioinformatics analyses using updated algorithms argue against that option. We have experimentally examined the mechanism underlying the interaction of this polypeptide with the PM. Data presented in this report show that the polycationic VP5 C-terminal tail is essential for the PM targeting of this polypeptide. Additionally, we demonstrate that VP5 specifically binds different phosphoinositide (PIP) species, and that this interaction is mediated by its electropositive C-terminal tail. Finally, we show that VP5 is a key element on IBDV cell-to-cell dissemination, and that ablation of its C-terminal tail causes a significant plaque size reduction.

## Materials and Methods

### Cells, viruses, infections, and transfections

HeLa (human epithelial cervical cancer cells, ATCC number CCL-2TM), DF-1 (spontaneously transformed chicken embryo fibroblasts, ATCC number CRL-12203), BSC40 (African green monkey kidney cells, ATCC number CRL-2761), and QM7 (quail muscle myoblasts, ATCC number CRL-1962) were grown in Dulbecco′s modified Eagle's medium (DMEM) supplemented with penicillin (100 U/ml), streptomycin (100 mg/ml) and 5% fetal calf serum (FCS) (Sigma).

Recombinant vaccinia viruses (VACV) were grown and titrated in BSC40 cells. Cultures were mock-infected or infected with the different viruses diluted in DMEM to the indicated multiplicity of infection (MOI). Derivatives of the IBDV Soroa strain, a cell-adapted serotype I virus, generated by reverse genetics were propagated as described [13]. IBDV titrations were performed in QM7 cells as described below (see IBDV plaque assay). Virus infections were performed by diluting virus stocks in DMEM. After washing with PBS, monolayers were incubated with virus suspensions for 1 h at 37°C. After virus adsorption, the medium was replaced with fresh DMEM supplemented with 2% FCS. In cells infected with recombinant VACVs, expression of isopropyl β-D-thiogalactosidase (IPTG)-inducible genes was triggered at the indicated times p.i. by adding IPTG (Apollo Scientific) to cell culture medium to reach a final concentration of 1 mM. Transfections were carried out on preconfluent HeLa cell monolayers with 100 ng of plasmid DNA per 10^5^ cells using lipofectamine 2000 (Invitrogen).

### Construction of plasmids expressing green fluorescent protein (GFP)-VP5 fusion proteins

Plasmid pEGFP/VP5 was generated by cloning a DNA fragment generated by PCR using the oligonucleotides 5’-GCGCGCGAATTCTATGGTTAGTAGAGATCAGACA and 5’-GCGCGCGGATCCCTCAGGCTTCCTTGGAAGGTC and using the previously described pBSK/VP5 plasmid as template [12]. This fragment was digested with EcoRI/BamHI and cloned into pEGFP-C1 (Clontech) digested with the same enzymes. GFP/CT122-145 was generated by cloning a DNA fragment generated by the annealing of oligonucleotides 5’-AATTCAAAGCACACCAGCTGGTGGAGGCTGTGCACCAAGAGGCACCACAAGAGGAGGGACCTGCCCAGGAAGCCCGAGTAAG and 5’-GATCCTTACTCGGGCTTCCTGGGCAGGTCCCTCCTCTTGTGGTGCCTCTTGGTGCACAGCCTCCACCAGCTGGTGTGCTTTG into the pEGFP-C1 digested with EcoRI and BamHI. Plasmids were subjected to nucleotide analysis to assess the accuracy of inserted sequences.

### Generation of recombinant VACV

DNA fragments containing N-terminal Flag tagged VP5 gene versions were generated by PCR from pT7-SA-Rz [14] using the oligonucleotide 5’-CGCGCTCGAGCATATGGATTACAAGGATGACGACGATAAGGTTAGTAGAGATCAGACAAACG as forward primer, which includes the FLAG (DYKDDDDK) coding sequence, preceded by an in-frame ATG, immediately upstream of the VP5 ORF lacking the initial ATG, and 5’-GCGCGGATCCTCACTCAGGCTTCCTTGGAAGGTC, 5’- GCGCGGATCCTCAAAGGTCACGGCGTTTATGGTG, 5’- GCGCGGATCCTCAATGGTGCCGTTTAGTGCATAAAC or 5’- GCGCGGATCCTCAGCATAA ACGCCACCAGGAAGTG as reverse primers, respectively. DNA fragments were digested with NdeI and BamHI, and cloned into the insertion/expression pVOTE.2 vector [15] digested with the same enzymes, generating plasmids pVOTE.2/FVP5, pVOTE.2/FVP5Δ5, pVOTE.2/FVP5Δ10 and pVOTE.2/FVP5Δ15, respectively. Plasmids pVOTE.2/FVP5M1, pVOTE.2/FVP5M2, pVOTE.2/FVP5M3 and pVOTE.2/FVP5M4 were generated following a similar cloning strategy. In this case, mutant VP5 sequences were generated by gene synthesis (GeneScript). FVP5 sequences including the following amino acid substitutions: A^394^→G, A^395^→C, C^397^→G, G^398^→C and G^399^→T for FVP5M2; A^406^→G, A^407^→C, C^409^→G, G^410^→C, C^411^→T, C^412^→G and G^413^→C for FVP5M3; A^424^→G, G^425^→C, G^426^→A, A^427^→G and A^428^→C for FVP5M4; and all the previous substitutions for FVP5M1. All plasmids were subjected to nucleotide sequencing to assess the accuracy of inserted sequences, and then used to generate the following recombinant VACVs: VT7/FVP5, VT7/FVP5Δ5, VT7/FVP5Δ10, VT7/FVP5Δ15, VT7/FVP5M1, VT7/FVP5M2, VT7/FVP5M3, and VT7/FVP5M4, respectively. Generation of recombinant VACVs was performed by infecting BSC40 cells with the VT7LacOI virus [15], followed by transfection with the corresponding pVOTE.2 plasmid derivatives. Selection and amplification of recombinant VACVs were carried out as described [15].

### Expression and purification of Flag-tagged VP5 recombinant proteins

Recombinant FVP5, FVP5Δ15, and FVP5M1 genes were excised from the corresponding pVOTE.2 derivatives by digestion with NdeI and BamHI, and cloned into the prokaryotic expression vector pRSETA (Life Technologies) digested with the same enzymes. Noteworthy, restriction of pRSETA with NdeI and BamHI removes the initiator ATG, the 6xhis tag, the Express epitope and the enterokinase cleavage recognition sequence from the plasmid. Accordingly, recombinant FVP5 genes were placed immediately downstream from the ribosome binding site, thus allowing the expression of recombinant proteins lacking heterologous N- or C-terminal sequences. The corresponding pRSETA derivatives were used to transform BL21(DE3)pLysS *E*. *coli* competent cells (Life Technologies). Recombinant protein expression was performed at 18°C for 16 h following supplier′s instructions. Bacterial pellets were resuspended in lysis buffer (50 mM Tris pH 8, 150 mM NaCl, 0.1% Igepal, 50 μg/ml lysozyme, and EDTA-free complete protease inhibitor cocktail from Roche). After 30 min at 37°C, samples were sonicated, supplemented with 5 units/ml of DNaseI, and MgSO_4_ to reach a final concentration of 10 mM, and further incubated for 15 min at 37°C. Thereafter, samples were subjected to centrifugation at 15,000xg for 15 min to eliminate large debris. Collected supernatants were subjected to a second round of centrifugation at 100,000xg for 1 h. The resulting supernatants were subjected to affinity purification using anti-FLAG M1 agarose affinity gel (Sigma) following the supplier′s instructions. Purified protein samples were analyzed by SDS-PAGE and Western blotting.

### Generation of IBDV mutant viruses

Mutagenesis was performed by PCR using the pT7-SA-Rz plasmid as template and the oligonucleotide pairs 5’-AACGCTATCATTGATAGTTAGTAGAGATCAG and 5’-CTGATCTCTACTAACTATCAATGATAGCGTT, 5’- CATAAACGCCGTGACCTTCCAAGGTAGCCTGAGTGAACTGACAGATG and 5’- CATCTGTCAGTTCACTCAGGCTACCTTGGAAGGTCACGGCGTTTATG, 5’- GTTTATGCACT AAACGGCACCATTAACGCCGTGACCTTCCAAG and 5’- CTTGGAAGGTCACGGCGTTAATGGTGCCGTTTAGTGCATAAAC, 5’- CCTGGTGGCGTTTATGCACTTAACGGCACCATAAACGCCGTG and 5’- CACGGCGTTTATGGTGCCGTTAAGTGCATAAACGCCACCAGG to generate the following plasmids: pT7-SA-Rz/VP5KO, pT7-SA-Rz/VP5Δ3CT, pT7-SA-Rz/VP5Δ10CT, pT7-SA-Rz/VP5Δ14CT, respectively. These plasmids were used to obtain and amplify the IBDV VP5KO, VP5Δ3CT, VP5Δ10CT and VP5Δ14CT mutants using a previously described reverse genetics protocol [14].

### Confocal laser scanning microscopy (CLSM) analysis

Cells seeded onto glass coverslips were infected with indicated viruses or transfected with plasmids expressing different GFP fusion proteins. At the indicated times p.i. or post-transfection, coverslips were washed with PBS, fixed with 4% paraformaldehyde (Sigma) for 30 min, and then permeabilized by incubation with PBS containing Triton X-100 (Sigma) 0.5% for 5 min (transfections), or fixed with methanol for 5 min at -20°C, and air dried (infections). Coverslips were blocked for 20 min using a solution of PBS containing 5% FCS. Thereafter, samples were incubated for 1 h with mouse anti-FLAG (OriGene) or anti-GFP monoclonal antibodies (mAb), or rabbit anti-VP5, diluted in PBS supplemented with 1% FCS. Coverslips were repeatedly washed in PBS and incubated for 1 h with the appropriate secondary antibody, either goat anti-mouse or-rabbit Ig coupled to Alexa-488, diluted in PBS supplemented with 1% FCS. Cell nuclei were stained with 2-(4-amidinophenyl)-1H-indole-6-carboxamidine (DAPI; Sigma) diluted in PBS for 30 min. All incubations were performed at room temperature (RT). Finally, coverslips were air-dried, and mounted with ProLong antifading reagent (Invitrogen). Samples were visualized by epifluorescence using a Leica TCS-Sp5 microscope confocal system. Fluorescent signals detected by CLSM were recorded separately by using appropriate filters. Images were captured using the LAS-AF v.2.6.0 software package (Leica Microsystems). The anti-VP5 serum was obtained by immunizing New Zealand rabbits with affinity-purified Flag-tagged VP5 following a previously described protocol [16]. VP5 subcellulat distribution analyses were performed using the Image J program (http://imagej.nih.gov/ij/) on images corresponding to basal confocal sections from single cells stained with either anti-Flag or-VP5 serum. Cells showing more than 70% of the total VP5-specific signal at either compartment were scored as having a predominantly plasma membrane (P) or cytoplasmic (C) distribution. The remaining cell fraction was considered as exhibiting a mixed (Mx) plasma membrane/cytoplasmic distribution. Statistical analyses were performed with data from three independent experiments encompassing over three hundred single cell images per case.

### SDS-PAGE and Western Blot

Protein extracts were mixed with Laemmli′s sample buffer and heated at 95°C for 5 min. Electrophoreses were performed on 15% SDS-PAGE gels. For Western blot analysis, after electrophoresis, proteins were electroblotted onto nitrocellulose membranes (Bio-Rad). Before incubation with specific antibodies, membranes were blocked by incubation with 5% non-fat dry milk in PBS for 1 h at RT. Western blots were carried out using a mouse monoclonal antibody anti-FLAG (OriGene), or rabbit anti-VP5 diluted in blocking solution, respectively. After incubation with primary antibodies, membranes were thoroughly washed with PBS, incubated with goat anti-mouse Ig-Peroxidase conjugate (Sigma) or goat anti-rabbit Ig-Peroxidase conjugate (Sigma) diluted in blocking solution. Immunoreactive bands were detected by ECL Plus Immunoblotting Detection Reagents (GE Healthcare).

### Lipid overlay assay

Hydrophobic membranes spotted with 100 pmol of different phospholipids or with a concentration gradient of all eight phosphoinositides were purchased from Echelon Biosciences. Membranes were blocked for 90 min at RT in blocking buffer (BBF; PBS supplemented with 5% lipid-free BSA, 0.1% Tween-20, 3 mM CaCl_2_), and then incubated for 90 min with 5 μg/ml of affinity purified Flag-tagged fusion proteins diluted in BBF. Thereafter, membranes were washed with BBF and incubated for 1 h at RT with a mouse anti-FLAG mAb. Membranes were washed with BBF, and then incubated for 1 h with goat-anti mouse Ig-Peroxidase conjugate antibody (Sigma). After washing with BBF, bound proteins were detected by ECL. Incubations and washes were performed at RT. Membranes incubated with the different proteins were washed for the same length of time and detected with the same exposure time in each experiment.

### IBDV plaque assay

Monolayers of QM7 cells grown in 6-well culture plates were infected with serial dilutions of viruses under analysis. After virus adsorption, monolayers were covered with a mixture (v:v) of 2xDMEM and 1.4% agar. Cell cultures were maintained for 72 h at 37°C. Thereafter, the semisolid medium was carefully removed, and after washing with ice-cold PBS, monolayers were fixed with methanol-acetone (1:1) for 2 min at -20°C. Fixed monolayers were washed twice with PBS, and then incubated for 30 min at RT with PBS supplemented with 3% FCS. After this period, monolayers were incubated with rabbit anti-VP2 serum [17] diluted in PBS supplemented with 3% FCS for 90 min at RT. Monolayers were washed twice with PBS, and incubated with goat anti-rabbit Ig-Peroxidase conjugate antibody in PBS 3% FCS for 1 h. After gentle wash with PBS, monolayers were incubated with 3,3,9-diaminobenzidine (Sigma) substrate solution. The statistical analysis was carried out using the GraphPad Prism software 4.0 (GraphPad Software Inc.). A Kruskal-Wallis test was performed followed by Dunn′s multiple comparison tests between lysis plaque areas detected in monolayers infected with the different mutant viruses against those generated by the wild type virus. Presented data correspond to three independent experiments totalizing a minimum of 750 plaques for each virus.

## Results

### The polycationic VP5 C-terminal end is sufficient to drive the GFP reporter to the PM

Although accumulation of VP5 at the PM of infected cells has been documented by several reports from different laboratories, the mechanism(s) used by this protein to reach this compartment remained to be experimentally explored. The identification of a mildly hydrophobic stretch within the central region of this polypeptide (residues 69 to 88) as a transmembrane domain by two servers, i.e. TM pred [18] and TopPred [19], led us to hypothesize that VP5 might behave as a type II transmembrane polypeptide [12]. However, subsequent topological predictions performed with programs using updated algorithms, i.e. SPOCTOPUS [20], PHOBIUS [21], DAS [22], and HMMTOP [23], failed to identify the presence an amphipathic α-helix that might act as a transmembrane region (data not shown). This contradicting information led us to analyze other alternatives to explain the PM targeting of this polypeptide.

**Fig 1:**
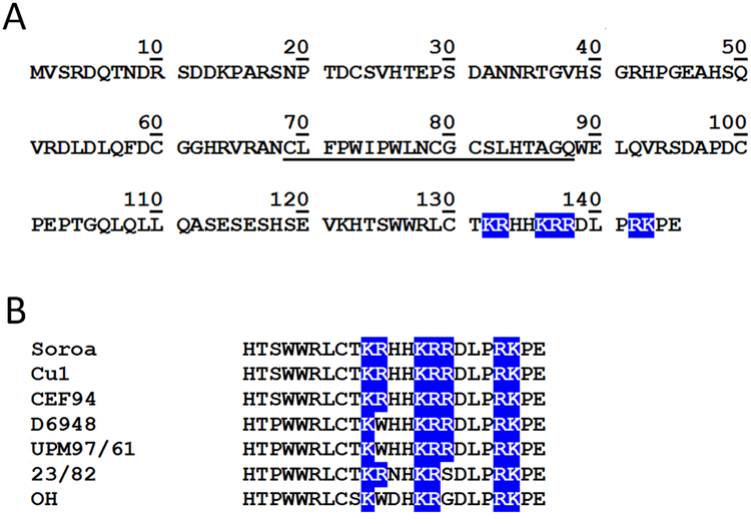
VP5 amino acid sequence. **A.** Deduced VP5 sequence corresponding to the IBDV Soroa strain. Positively charged amino acid residues at the C-terminal are highlighted in blue. Residues corresponding to the putative transmembrane domain are underlined. **B.** Multiple sequence alignment of the 23 VP5 C-terminal residues from classical virulent serotype I Soroa, CEF94 (accession number: AF194428), and Cu1 (D00867); very virulent serotype I D6948 (AF240686) and UPM97/61 (AF247006); and avirulent serotype II 23/82 (AF362773) and OH (IBU30818) strains. Conserved electropositive residues are highlighted in blue.

Inspection of the VP5 sequence revealed the presence of a polycationic C-terminal region spanning amino acid residues 132–143 containing three closely spaced clusters formed by two or three consecutive basic amino acid residues, namely ^132^KR^133^, ^136^KRR^138^ and ^142^RK^143^ (Fig 1A). A comparison of VP5 sequences from classical and very virulent serotype I and avirulent serotype II IBDV strains indicates that this region is fairly well conserved (Fig 1B). A wide variety of cellular proteins employ surface exposed positively charged domains to interact with anionic lipids anchored to the inner PM leaflet [24]. To explore whether the VP5 C-terminal tail might be involved in PM tropism, a sequence coding for the 24 VP5 C-terminal residues was fused in-frame to the 3′ end of the GFP ORF. This construct was named GFP/CT122-145. Two additional plasmids were used as control for subsequent experiments, i.e. pGFP/VP5 expressing the EGFP gene fused to the VP5 ORF lacking the initiation ATG codon (GFP/VP5), and pEGFPC1 expressing EGFP gene (GFP).

**Fig 2:**
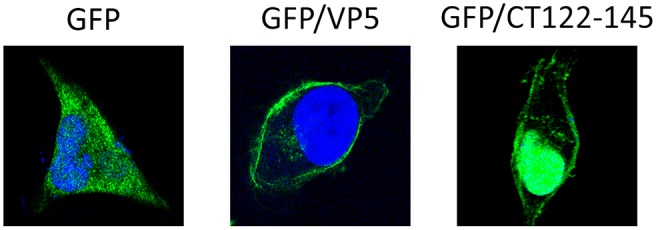
GFP PM targeting mediated by the 24 VP5 C-terminal residues. HeLa cells were transfected with plasmids encoding GFP (GFP), GFP fused to the VP5 ORF (VP5/GFP) or GFP fused to the 24 VP5 C-terminal residues (GFP/CT122-145). At 12 h post-transfection cultures were fixed and processed for IF using a mouse anti-GFP mAb followed by incubation with goat anti-mouse coupled to Alexa-488. Nuclei were stained with DAPI. Cells were visualized by CLSM. Fluorescence signals were recorded separately by using appropriate filters. Presented images correspond to confocal sections showing the overlay of the two fluorescence signals, and are characteristic examples of the GFP-specific immunofluorescence observed in cells transfected with the different plasmids.

Plasmids harboring the different constructs were transfected to preconfluent HeLa cell monolayers. At 12 h post-transfection cultures were fixed, processed for IF using a mouse anti-GFP mAb, and visualized by CLSM. As shown in Fig 2, the GFP protein showed a diffuse cytoplasmic/nuclear signal. As expected, the GFP-VP5 polypeptide accumulated at the PM. Noteworthy, the GFP/CT122-145 IF signal was specifically found to accumulate at both the PM and nuclei of transfected cells, thus indicating that this region is sufficient for an efficient PM targeting. PM targeting motifs resemble nuclear localization sequences [25]. Furthermore, it has been shown that the polybasic C-terminal tails from some small GTPases, e.g. Rit and Rin, drive GFP fusion proteins to both PM and nuclei [26]. Accordingly, the presence of the GFP/CT122-145 fusion protein at cell nuclei was not an unexpected finding.

### VP5 C-terminal tail ablation abolishes PM targeting

**Fig 3:**
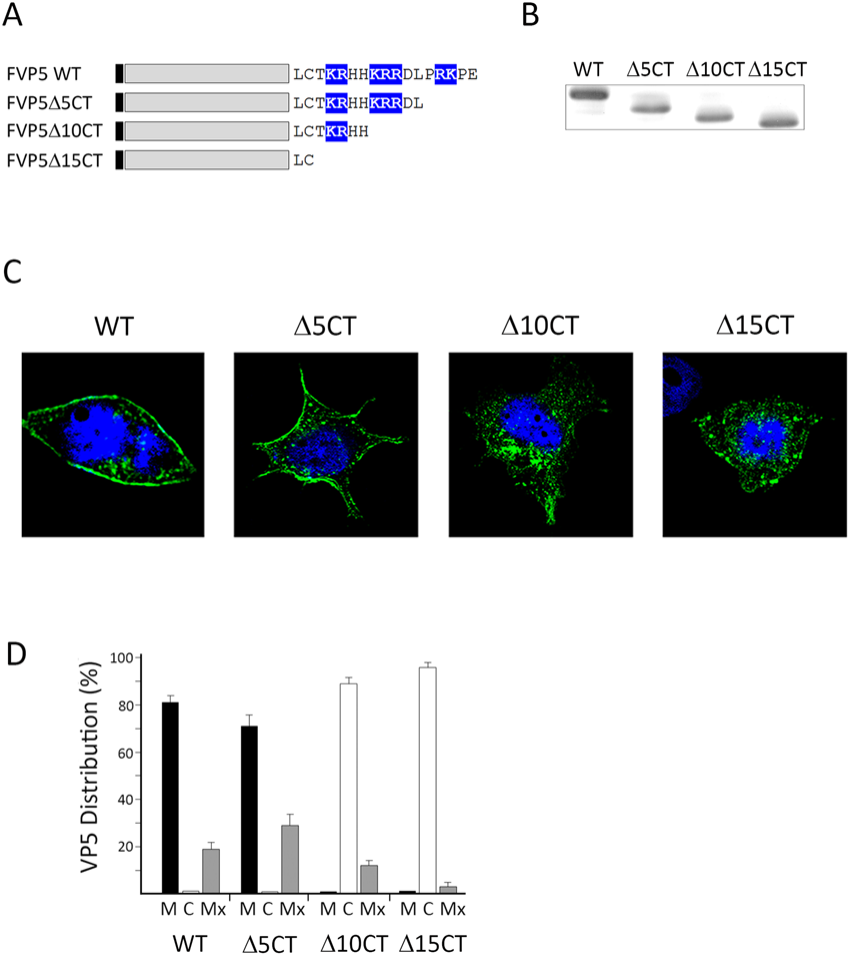
Effect of C-terminal deletions on VP5 PM targeting. A. C-terminal deleted VP5 polypeptides. Diagram depicting the wild type Flag-tagged VP5 protein (FVP5WT) and versions of the protein lacking either 5 (Δ5CT), 10 (Δ10CT) or 15 (Δ15CT) C-terminal residues expressed by recombinant VACVs used in this analysis. The black box indicates the position of the Flag tag. Positively-charged amino acid residues at the C-terminal are highlighted in blue. B. Protein expression analysis. Samples from cells infected with recombinant VACVs expressing the different proteins were subjected to SDS-PAGE and Western blot analysis using an anti-Flag mAb. Subcellular VP5 distribution. C. Single cell images. Coverslips containing cells infected with the different recombinant VACVs were processed for IF using a mouse anti-Flag mAb followed by incubation with goat anti-mouse coupled to Alexa-488 (Green). Nuclei were stained with DAPI (Blue). Cells were visualized by CLSM. Fluorescence signals were recorded separately by using appropriate filters. Images correspond to confocal sections showing the overlay of the two fluorescence signals, and are characteristic examples of the GFP-specific immunofluorescence observed in cells infected with the different recombinant VACVs. D. Statistical analysis. Cells showing more than 70% of the total Flag-specific signal at the plasma membrane or the cell cytoplasm were scored as having a predominant plasma membrane (P) or cytoplasmic (C) distribution. The remaining cell fraction was considered as exhibiting a mixed (Mx) plasma membrane/cytoplasmic distribution. Analyses were performed with data collected from three independent experiments using a total of over three hundred single cell images.

Data described above suggested the involvement of the VP5 C-terminal tail in PM targeting. To further analyze this hypothesis, three recombinant VP5 genes encoding polypeptides lacking 5 (FVP5Δ5CT), 10 (FVP5Δ10CT) and 15 (FVP5Δ15CT) C-terminal VP5 residues, respectively, were constructed. To facilitate their detection, a FLAG tag was fused to the N-termini of the recombinant polypeptides. These constructs were used to generate recombinant VACVs. The resulting recombinant VACVs were termed VT7/FVP5Δ5CT, VT7/FVP5Δ10CT and VT7/FVP5Δ15CT, respectively. A fourth recombinant VACV, VT7/FVP5, expressing the full length Flag-tagged VP5 protein was also generated and used as a control for subsequent experiments. A cartoon depicting the structure of the described constructs is shown in Fig 3A.

To analyze the expression and subcellular distribution of the mutant FVP5 polypeptides, HeLa cell monolayers were infected with the corresponding recombinant VACVs. At 6 h p.i. IPTG was added to cell media to trigger the expression of recombinant FVP5 genes. Cultures were maintained for 12 h and then either harvested and used for Western blot analysis or processed for IF using a mouse anti-FLAG mAb and visualized by CLSM.

Western blot analysis revealed that, as expected, VP5 C-terminal tail deletions result in the synthesis of recombinant FVP5 polypeptides with increasing electrophoretic motilities (Fig 3B). As shown in Fig 3C and 3D, FVP5 and FVP5Δ5CT showed a characteristic PM accumulation. FVP5Δ10CT was found mostly at the cytoplasm but retained some PM signal. Significantly, the FVP5Δ15CT protein, missing the three basic clusters, exhibited a punctuated pattern exclusively restricted to the cell cytoplasm. Taken together, these results indicate that the 15 VP5 C-terminal residues are critical for PM targeting.

### Electropositive C-terminal charge is essential for PM localization

**Fig 4:**
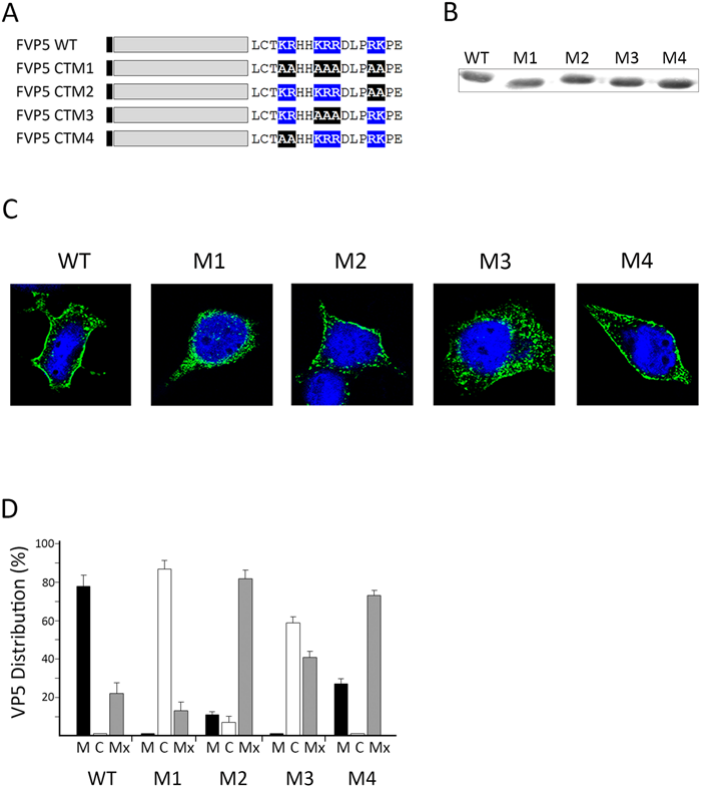
Contribution of the electropositive C-terminal charge to VP5 PM targeting. A. VP5 alanine replacement mutants. Diagram depicting Flag-tagged VP5 proteins expressed by the different recombinant VACVs used in this analysis. The M1 protein lacks the three cationic clusters whilst M2, M3, and M4 lack the ^132^KR^133^, the ^136^KRR^138^ or the ^142^RK^143^ clusters, respectively. A Flag-tagged version of the wild type protein (WT) was used as control. The black box indicates the position of the Flag tag. Positively charged amino acids at the VP5 C-terminus are highlighted in blue, and the replacing alanine residues in black. B. Protein expression analysis. Samples from cells infected with recombinant VACV expressing the different proteins were subjected to SDS-PAGE and Western blot analysis using anti-Flag mAbs. Subcellular VP5 distribution. C. Single cell images. Coverslips containing cells infected with the different recombinant VACVs were processed for IF using a mouse anti-Flag mAb followed by incubation with goat anti-mouse coupled to Alexa-488 (Green). Nuclei were stained with DAPI (Blue). Cells were visualized by CLSM. Fluorescence signals were recorded separately by using appropriate filters. Images correspond to confocal sections showing the overlay of the two fluorescence signals, and are characteristic examples of the GFP-specific immunofluorescence observed in cells infected with the different recombinant VACVs. D. Statistical analysis. Cells showing more than 70% of the total Flag-specific signal at the plasma membrane or the cell cytoplasm were scored as having a predominant plasma membrane (P) or cytoplasmic (C) distribution. The remaining cell fraction was considered as exhibiting a mixed (Mx) plasma membrane/cytoplasmic distribution. Analyses were performed with data collected from three independent experiments using a total of over three hundred single cell images.

Previous results suggested that the interaction of VP5 with the plasmalemma involves the establishment of an electrostatic interaction between the VP5 polybasic C-terminal tail and anionic PM lipids. Provided that was the case, the reduction of the net charge of the VP5 C-terminal tail should significantly affect PM targeting. To test this hypothesis, four Flag-tagged VP5 mutant versions, in which cationic clusters were selectively replaced by non-polar alanine (A) residues, were engineered. A diagram depicting the configuration of the C-terminal tails of the different mutants is shown in Fig 4A. The FVP5CTM1 recombinant gene lacks the three cationic clusters. The FVP5CTM2, FVP5CTM3, and FVP5CTM4 polypeptides lack the ^132^KR^133^, the ^136^KRR^138^ or the ^142^RK^143^ cluster, respectively. The described constructs were used to generate inducible recombinant VACVs. The resulting viruses were employed to infect HeLa cells. The electrophoretic mobility and the subcellular distribution of the different FVP5 mutant versions were analyzed by Western blot and CLSM analysis. VT7/FVP5, expressing the full length Flag-tagged VP5 protein was used as a control for these experiments.

The Western blot analysis showed the existence of minor variations of the electrophoretic profiles of the different FVP5 mutant proteins (Fig 4B). As shown in Fig 4C and 4D, FVP5CTM1 and FVP5CTM3 polypeptides did not significantly accumulate at the PM whilst FVP5CTM2 and FVP5CTM4 exhibited predominant mixed PM/cytosol distributions. These results unambiguously show that lessening the net electropositive charge of the VP5 C-terminal tail causes a major effect on PM targeting, thus complementing and confirming data gathered with VP5 C-terminal deletion mutants.

### VP5 binds different phosphoinositide species through its C-terminal tail

**Fig 5:**
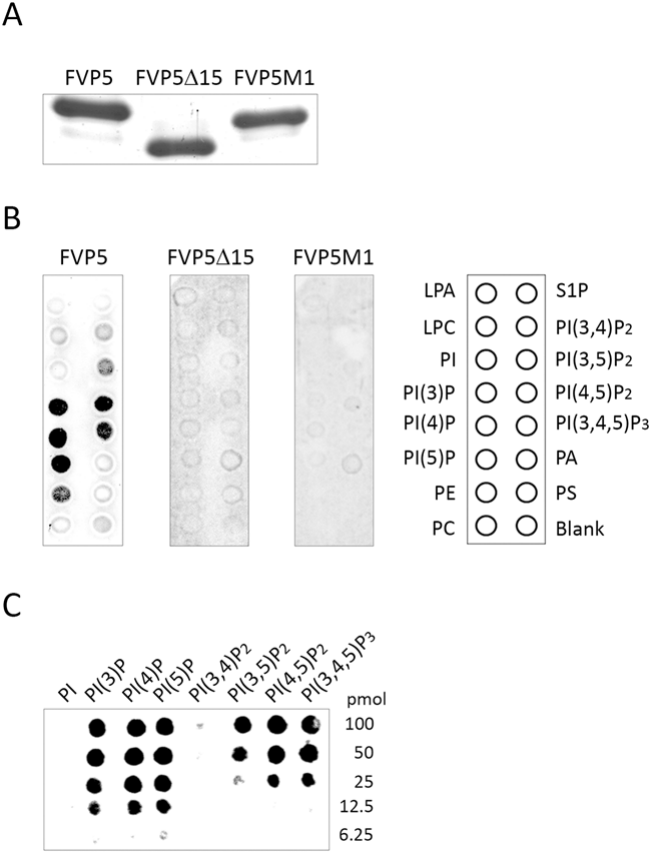
The positively charged VP5 C-terminal domain is required for binding to PIPs. A. Affinity purified VP5 polypeptides. The wild type (FVP5) and C-terminal mutant (FVP5Δ15CT and FVP5CTM1) proteins used for this analysis were expressed in *E*. *coli* and purified by affinity chromatography using anti-Flag agarose. Purified protein samples were analyzed by SDS-PAGE followed by Coomasie blue staining. B. Protein lipid overlay assay. Membranes spotted with 100 pmol of various lipids were incubated with 5μg/ml of the different proteins. Bound proteins were detected using an anti-Flag mouse mAb, followed by incubation with goat-anti mouse Ig coupled to peroxidase and developed with ECL. The right panel indicates the positions of spots corresponding to the different lipids. PA, phosphatidic acid; PC, phosphatidylcholine; PE, phosphatidylethanolamine; PI, phosphatidylinositol; PI(3)P, PI(3) phosphate; PI(4)P, PI(4) phosphate; PI(5)P, PI(5) phosphate; PI(3,4)P_2_, PI(3,4) bisphosphate; PI(3,5)P_2_, PI(3,5) bisphosphate; PI(4,5)P_2_, PI(4,5) bisphosphate; PI(3,4,5)P_3_, PI(3,4,5) trisphosphate; PS, phosphatidylserine; S1P, sphingosine-1-phosphate. C. FVP5 PIP binding. Membranes spotted with a concentration gradient of all eight phosphoinositides were incubated with FVP5. Overlay assays were performed as described above. PIP amounts (pmol) are indicated.

In view of results described above, it was important to assess the capacity of the VP5 polypeptide to interact with membrane lipids. For this, affinity-purified FVP5 protein (Fig 5A) was incubated with lipid arrays containing biologically relevant lipids, including phosphatidylinositol (PI) and its seven phosphorylated derivatives (phosphoinositides, PIP), lysophosphatidic acid (LPA), lysophosphocholine (LPC), phosphatidylethanolamine (PE), phosphatidylcholine (PC), sphingosine 1-phosphate (S1P), phosphatidic acid (PA) and phosphatidylserine (PS), found at the cytosolic face of membranous cell compartments [27]. As controls for these experiments, assays were also performed with affinity-purified FVP5Δ15CT and FVP5CTM1 mutant proteins (Fig 5A).

As shown in Fig 5B, the assays revealed a strong binding of the FVP5 polypeptide to the three monophosphate PIP species, i.e. PI 3-phosphate (PI(3)P), PI 4-phosphate (PI(4)P) and PI 5-phosphate (PI(5)P). A less efficient binding to PI(4,5) bisphosphate (PI(4,5)P_2_) and PI(3,4,5) trisphosphate (PI(3,4,5)P_3_) was also detected. Finally, much weaker binding signals were detected with PI(3,4) disphosphate (PI(3,4)P_2_) and PE. In contrast, significant lipid binding was not detected with FVP5Δ15 and FVP5CTM1 proteins, thus showing that the electropositive VP5 C-terminal domain is required for PIP binding. Accordingly, this protein region is henceforth described as the VP5 PIP-binding domain.

Array strips spotted with decreasing lipid amounts (100–6.25 pmol/spot) were used to comparatively assess the affinity of the protein for different PIPs. As shown in Fig 5C, VP5 exhibited a similar affinity for the three monophosphate PIP species, giving a significant signal with spots containing 12.5 pmols of these lipids. Significant binding to PI(3,5P)_2_, PI(4,5)P_2_, or PI(3,4,5)P_3_ was only found at higher (≥25 pmols/spot) lipid concentrations. The interaction with either PI(3,4)P_2_ or PI was negligible. These results indicate that VP5 shows a preference for monophosphate PIP species.

### Ablation of the PIP-binding domain abolishes VP5 PM localization in IBDV-infected cells

**Fig 6:**
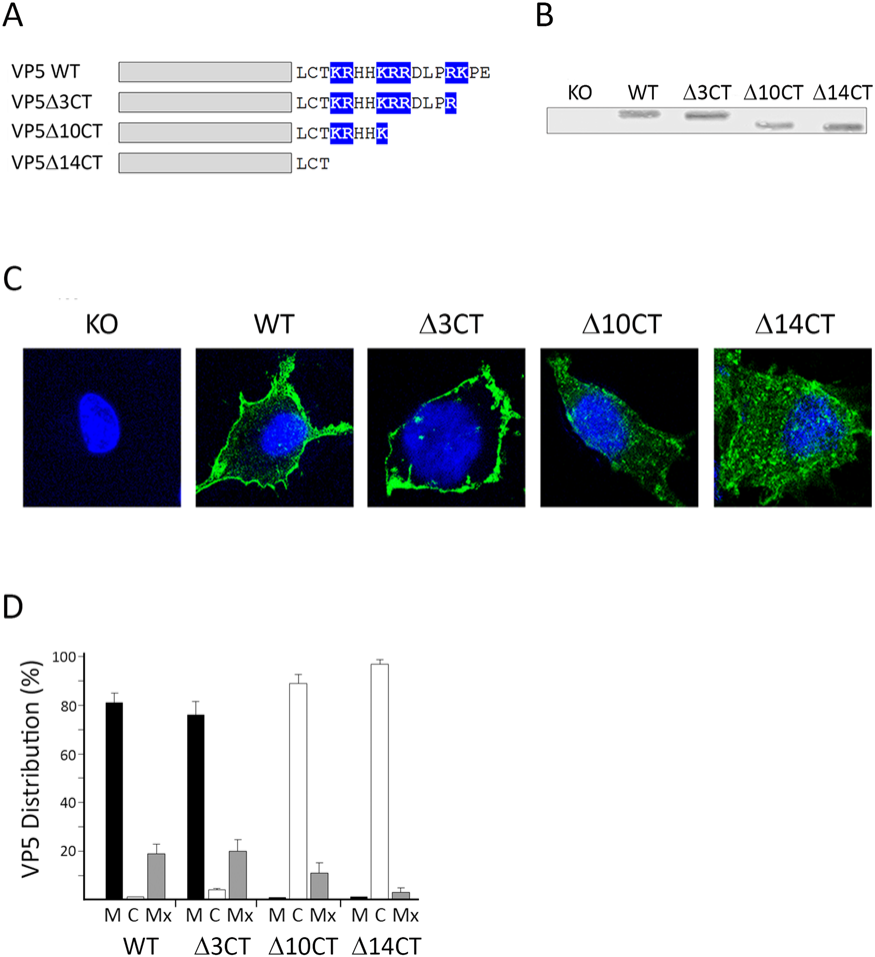
C-terminal ablation abolishes PM VP5 targeting in IBDV-infected cells. A. C-terminal VP5 ablation IBDV mutants. Diagram depicting the VP5 proteins expressed by IBDV mutants lacking 3 (Δ3CT), 10 (Δ10CT), and 14 (Δ14CT) residues used in this analysis. The wild type virus (WT) and a VP5 knockout mutant (KO) were used as controls. Positively charged amino acids at the C-terminal are highlighted in blue. B. VP5 expression. Samples from cells infected with different viruses were subjected to SDS-PAGE and Western blot analysis using anti-VP5 serum. Subcellular VP5 distribution. C. Single cell images. Coverslips containing cells infected with the different viruses were processed for IF using rabbit anti-VP5 serum followed by incubation with goat anti-rabbit coupled to Alexa-488. Nuclei were stained with DAPI. Cells were visualized by CLSM. Fluorescence signals were recorded separately by using appropriate filters. Images correspond to confocal sections showing the overlay of the two fluorescence signals. Images correspond to confocal sections showing the overlay of the two fluorescence signals, and are characteristic examples of the VP5-specific immunofluorescence observed in cells infected with the different viruses. D. Statistical analysis. Cells showing more than 70% of the total VP5-specific signal at the plasma membrane or the cell cytoplasm were scored as having a predominant plasma membrane (P) or cytoplasmic (C) distribution. The remaining cell fraction was considered as exhibiting a mixed (Mx) plasma membrane/cytoplasmic distribution. Analyses were performed with data collected from three independent experiments using a total of over three hundred single cell images.

Experiments described above were carried out using recombinant VP5 genes expressed in a heterologous context. Indeed, data obtained from recombinant expression systems should be treated with some caution. It was therefore important to investigate the effect of mutations affecting the PIP-binding domain within the context of the IBDV infection. To perform this analysis, three IBDV mutants, namely IBDV_VP5Δ3CT, IBDV_VP5Δ10CT, and IBDV_VP5Δ14CT, expressing truncated versions of the VP5 polypeptides lacking 3, 10, and 14 VP5 C-terminal residues, respectively, were generated by reverse genetics (Fig 6A). The segment A of these three viruses contain a single nucleotide substitution A^489^→U, A^501^→U or A^522^→U, respectively, that convert the “AAA” codons coding for VP5 K^132^ and K^136^, or the “AAG” codon coding for K^142^, into “UAA” or “UAG” translation termination codons. Noteworthy, these substitutions result in the introduction of synonymous mutations within the overlapping ORF, thus not altering the amino acid sequence of the virus polyprotein. Two additional viruses, i.e. wild type IBDV (IBDV_WT), expressing an unmodified VP5 version of the IBDV Soroa strain (WT), and IBDV VP5 knockout (IBDV_VP5 KO), were also generated by reverse genetics and used as controls for subsequent experiments. IBDV_VP5 KO contains a single nucleotide substitution, i.e. segment A: G^98^→A, converting the VP5 “AUG” translation initiation codon into an “AUA” triplet, thus abolishing VP5 expression [8]. A diagram depicting the configuration of the C-terminal tails of the different mutants is shown in Fig 6A.

The described viruses were used to infect monolayers of DF1 cells at a multiplicity of infection of 3 plaque forming units/cell. At 24 h p.i., cultures were either harvested to assess the expression of the VP5 and VP3 IBDV-encoded proteins by Western blot or processed for IF using anti-VP5 serum.

The different VP5 mutant proteins showed the expected electrophoretic mobility differences (Fig 6B). Despite this, the VP5-specific bands detected in cells infected with the different VP5-expressing viruses showed similar relative intensities, thus indicating that the introduced mutations do not significantly affect the VP5 expression level. Indeed, VP5 expression was not detected in cells infected with the IBDV_VP5 KO mutant.

As expected, whilst the largest fraction of the VP5 protein encoded by IBDV_WT accumulated at the PM, no specific VP5 signal was detected in cells infected with the IBDV_VP5 KO mutant (Fig 6C and 6D). The polypeptide encoded by IBDV_Δ3CT gene was found to predominantly accumulate at the PM, thus closely resembling the behavior of the WT protein (Fig 6C and 6D). The protein encoded by IBDV_VP5Δ10CT exhibited a cytosolic localization similar to that observed with the recombinant FVP5Δ10CT protein (Fig 6C and 6D). Finally, the VP5 protein encoded by IBDV_VP5Δ14CT was found to accumulate almost exclusively at the cell cytoplasm (Fig 6C and 6D), thus mirroring what had been previously observed with the FVP5Δ15CT recombinant protein lacking the 15 C-terminal residues (Fig 3C and 3D). These results demonstrate that VP5 proteins encoded by the different IBDV mutants exhibit the expected subcellular distribution when expressed in avian cells during virus replication.

### Deletions affecting the PIP-binding domain reduce the IBDV cell-to-cell spreading capacity and extracellular virus yields

Unpublished data from our laboratory indicated that abrogation of VP5 expression causes a major reduction on the virus plaque size phenotype. Thus, in order to analyze the effect of the described VP5 C-terminal deletions on the ability of the virus to disseminate from cell-to-cell, we compared the plaque size phenotype of the different mutant viruses to that of the wild type and VP5 knockout viruses. For this, QM7 monolayers grown in 6-well culture plates were infected with serial dilutions of stocks corresponding to IBDV_WT, _VP5 KO, _VP5Δ3CT, _VP5Δ10CT, and _VP5Δ14CT viruses described above. After infection, monolayers were washed twice with PBS and then covered with DMEM supplemented with 0,75% agarose and 2% FCS. At 72 h p.i. the semisolid media was carefully removed, and cell monolayers processed for immunostaining using antibodies specifically recognizing the IBDV VP2 capsid polypeptide.

**Fig 7:**
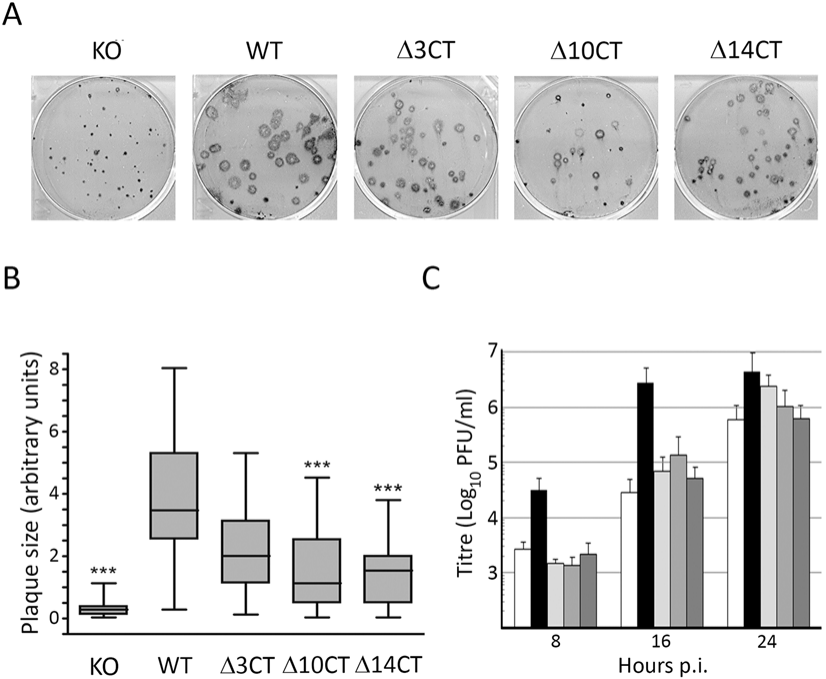
VP5 C-terminal ablation affects the IBDV plaque size phenotype. A. Plaque assays. QM7 cell monolayers were infected with the VP5 knockout mutant (KO) virus, the wild type (WT) virus and mutant IBDV viruses lacking 3 (Δ3CT), 10 (Δ10CT), and 14 (Δ14CT) VP5 C-terminal residues, respectively. After infection, monolayers were covered with semisolid medium. After three days, the medium was removed and virus-induced plaques were detected by immunostaining using serum specifically recognizing the VP2 IBDV capsid polypeptide. B. Statistical analysis. The statistical analysis was carried on images from immnunostained monolayers using the GraphPad Prism software 4.0. A Kruskal-Wallis test was performed followed by Dunn’s multiple comparison tests between lysis plaque areas detected in monolayers infected with the different mutant viruses and those generated by the wild type virus. Presented data correspond to three independent experiments totalizing 750 plaques for each virus. Significant differences (p<0.001) between KO, Δ10CT, Δ14CT and WT viruses are indicated with asterisks (***). C. Extracellular virus yields. QM7 cells were infected with the VP5 knockout mutant (white columns), wild type (black) and mutants viruses VP5Δ3CT (light grey), VP5Δ10CT (medium grey), and Δ14CT (dark grey), at an MOI of 3 PFU/cell. At 8, 16 and 24 h p.i. cultures were harvested and subjected to centrifugation at 15,000xg for 15 min to eliminate cells and large debris. The corresponding supernatants were used to determine extracellular virus titres. Presented data correspond to three independent experiments.

As shown in Fig 7A, lysis plaques formed by the VP5 knockout mutant (IBDV_VP5 KO) are remarkably smaller (over 14xfold) than those observed in cells infected with the wild type virus, thus showing that the blockade of VP5 expression caused by the elimination of its translation initiation codon results in a dramatic reduction of the cell-to-cell spreading capacity of the virus. Noteworthy, the three VP5 C-terminal truncation mutants also exhibited conspicuous reductions, ranging from 1.7–3.1xfold, on the average plaque diameter. Statistical analysis showed that plaque size reductions observed with the VP5Δ10CT and VP5Δ14CT mutants are statistically significant (p<0.001) (Fig 7B).

Notwithstanding the clonal origin of viruses used in our analyses, a fraction of abnormally small (≤5xfold smaller than the corresponding average size) plaques was found with all tested viruses. Intriguingly, plaque assays performed with WT virus clones picked up from either “normal” or “small” plaques showed the presence of the both described plaque populations (data not shown), thus the basis for this heterogeneity remains unknown.

The effect of the VP5 C-terminal truncations on extracellular virus yields was determined by titration of cell medium samples collected at different times p.i., i.e. 8, 12 and 24 h, from cultures infected with 3 PFU/cell of the different viruses under study. As shown in Fig 7C, the three analyzed C-terminal deletions cause a detectable reduction of extracellular virus yields, particularly conspicuous at intermediate times p.i., that in the case of VP5Δ10CT and VP5Δ14CT is comparable to that observed with the VP5 KO mutant.

Results described in this section demonstrate that ablation of PIP-binding domain significantly lessens the cell-to-cell spreading ability of the virus in tissue culture, thus mimicking the effect observed with the VP5 knockout mutant virus.

## Discussion

### The role of the VP5 C-terminal tail in PM targeting

The relative predominance of anionic lipid species at the inner PM leaflet provides this surface with an overall negative electrostatic environment that attracts proteins containing surface-exposed polycationic domains [27]. Indeed, electrostatic interactions between anionic lipids and surface exposed polycationic protein domains play a key role on the targeting of many proteins to the plasmalemma [24]. The presence of three positively charged clusters, ^132^KR^133^, ^136^KRR^138^ and ^142^RK^143^, within the C-terminal VP5 tail suggested that this region might be important for the interaction of this protein with the PM. Experiments performed with GFP/CT122-145, a GFP C-terminal fusion containing the 24 C-terminal VP5 residues, show that this region is sufficient to target GFP to the PM. Additionally, data gathered with recombinant Flag-tagged VP5 versions, harboring C-terminal deletions or amino acid substitutions, evidenced that the reduction of the net electropositive charge of the C-terminal domain either weaken or abolish the interaction of the protein with the PM. Identical effects were observed in cells infected with IBDV VP5 C-terminal ablation mutants generated by reverse genetics. Taken together these data demonstrates that the C-terminal domain is essential for the PM tropism of this protein, thus ruling out our previous hypothesis that VP5 might behave as a class II transmembrane polypeptide.

As expected for a functionally important domain, the VP5 C-terminal tail is well conserved amongst IBDV strains. A comparison of VP5 sequences from IBDV strains exhibiting different virulence levels showed the existence of only one conserved amino acid substitution, R^133^→W, differentiating classical virulent from very virulent serotype I strains [28]. Interestingly, this mutation lies within the first electropositive patch of the polycationic domain. Indeed, the potential contribution of this substitution on VP5 subcellular distribution, lipid interaction, and virus dissemination deserves a detailed analysis.

### VP5-PIP interaction

Data presented here show that, excluding PI(3,4)P_2_, VP5 efficiently binds PIPs, exhibiting a higher affinity for the three monophosphate species. Apart from PE which appears to be weakly recognized by VP5, interaction with other relevant anionic membrane lipids was not detected. This suggests that, in addition to the electrostatic charge, the structure of the VP5 C-terminal domain plays a critical role for PIP interaction.

Hitherto eleven PIP-binding modules have been described. Structural and functional characterization of these modules indicates that both the affinity and selectivity for different PIP species are precisely regulated by their 3D structure [29]. Interestingly, the PI(3)P-binding site from proteins containing one of these modules, i.e. the FYVE (Fab1,YGLO23, Vps27, and EEA1) module, is formed by the conserved (R/K)(R/K)HHCR sequence [30] closely resembling the ^132^KRHHKR^137^ stretch at the VP5 PIP-binding domain. The possible relevance of this similarity is unknown. Certainly, the resolution of the VP5 crystal structure would provide an important insight for the understanding of the mechanism regulating its interaction with PIPs. The finding that some proteins encoded by genes generated by overprinting mechanisms correspond to new protein folds [31,32] adds further interest to this task.

Although PIPs only represent a minor fraction of the total lipid content of the cytosolic leaflets of the PM and other intracellular membranes [27], they act as key elements in a plethora of cellular functions. PIPs act as precursors of second messengers in signal transduction and control, either directly or indirectly, a wide variety of pathways involved in cell survival, cytoskeletal dynamics, and vesicular traffic [33]. Indeed, there is growing evidence that modulation of PIP metabolism plays an important role in the pathogenicity of some intracellular pathogens, including viruses [34–36].

It has been recently described that VP5 interacts with the regulatory p85α subunit of the phosphatidylinositol 3-kinase (PI3K) leading to activation of the PI3K/Akt signaling pathway [7]. Our finding is, in principle, compatible with this observation. VP5 polypeptides bound to the cytosolic PM leaflet might act as docking platforms for PI3K via its interaction with the p85α subunit. This would provide the means to activate the PI3K/Akt signaling pathway in the absence of other stimuli. In this scenario, the selectivity of VP5 for monophosphate PIP species would restrict a possible competition for PI3K substrates, thus further facilitating the activation of this pathway.

Considering data presented here, is feasible to envisage that VP5 overexpression might cause a major deregulation of PIP-dependent pathways, including those controlling cytoskeletal dynamics [37]. This might explain our previous observation that expression of VP5 from recombinant VACV vectors alters cell morphology, promotes membrane ruffling, increases cell motility and eventually leads to cell lysis [12].

### IBDV cell-to-cell spread

Here, we show for the first time that the VP5 knockout causes an overwhelming reduction on the diameter of virus plaques. This finding further supports data described in several reports indicating that the VP5 polypeptide is involved in the release of the IBDV progeny, and provides direct evidence that VP5 is a key element in the IBDV dissemination. Our data, showing that viruses harboring on the VP5 PIP-binding domain deletions exhibit significant plaque size reductions indicate that the VP5-PIP interaction(s) is an important element for efficient IBDV propagation.

Specific information concerning Birnavirus egress mechanisms is not available as yet. Birnavirus particles are naked icosahedrons. Consequently, it has customarily assumed that, as other viruses lacking membranous envelopes, their release is concomitant to the destruction of the infected cell. However, there is growing evidence that a variety of naked viruses, including adeno-, polyoma-, and picornaviruses, use alternative mechanisms affording efficient non-lytic cell-to-cell spreading before cell membrane obliteration takes place [37–41]. Data presented here suggest the possibility that IBDV might also use a VP5-dependent non-lytic cell-to-cell spreading mechanism. The loss of such mechanism might lay behind the drastic reduction of both plaque size and virulence observed with VP5 KO viruses.
